# RSK inhibitor BI-D1870 inhibits acute myeloid leukemia cell proliferation by targeting mitotic exit

**DOI:** 10.18632/oncotarget.27630

**Published:** 2020-06-23

**Authors:** Hee-Don Chae, Ritika Dutta, Bruce Tiu, Fieke W. Hoff, Benedetta Accordi, Valentina Serafin, Minyoung Youn, Min Huang, Nathan Sumarsono, Kara L. Davis, Norman J. Lacayo, Martina Pigazzi, Terzah M. Horton, Steven M. Kornblau, Kathleen M. Sakamoto

**Affiliations:** ^1^Department of Pediatrics, Stanford University School of Medicine, Stanford, CA, USA; ^2^Department of Pediatric Oncology/Hematology, Beatrix Children’s Hospital, University Medical Center Groningen, University of Groningen, Groningen, The Netherlands; ^3^Department of Women’s and Children’s Health, Onco-Hematology Clinic, University of Padova, Padova, Italy; ^4^Texas Children's Cancer and Hematology Centers, Baylor College of Medicine, Houston, TX, USA; ^5^Department of Leukemia, The University of Texas MD Anderson Cancer Center, Houston, TX, USA

**Keywords:** acute myeloid leukemia, BI-D1870, RSK, vincristine, spindle assembly checkpoint

## Abstract

The 90 kDa Ribosomal S6 Kinase (RSK) drives cell proliferation and survival in cancers, although its oncogenic mechanism has not been well characterized. Phosphorylated level of RSK (T573) was increased in acute myeloid leukemia (AML) patients and associated with poor survival. To examine the role of RSK in AML, we analyzed apoptosis and the cell cycle profile following treatment with BI-D1870, a potent inhibitor of RSK. BI-D1870 treatment increased the G2/M population and induced apoptosis in AML cell lines and patient AML cells. Characterization of mitotic phases showed that the metaphase/anaphase transition was significantly inhibited by BI-D1870. BI-D1870 treatment impeded the association of activator CDC20 with APC/C, but increased binding of inhibitor MAD2 to CDC20, preventing mitotic exit. Moreover, the inactivation of spindle assembly checkpoint or MAD2 knockdown released cells from BI-D1870-induced metaphase arrest. Therefore, we investigated whether BI-D1870 potentiates the anti-leukemic activity of vincristine by targeting mitotic exit. Combination treatment of BI-D1870 and vincristine synergistically increased mitotic arrest and apoptosis in acute leukemia cells. These data show that BI-D1870 induces apoptosis of AML cells alone and in combination with vincristine through blocking mitotic exit, providing a novel approach to overcoming vincristine resistance in AML cells.

## INTRODUCTION

Acute myeloid leukemia (AML) is a genetically and phenotypically heterogeneous hematologic malignancy characterized by the accumulation of immature myeloid blasts with resultant peripheral blood cytopenia [1, 2]. Even with traditional intensive chemotherapy regimens, AML is the most common cause of leukemia death [3], with a 5-year survival of less than 60% in younger AML patients and less than 10% in the elderly AML patients [2, 4]. Furthermore, AML survivors often experience chronic health conditions as a result of their treatment that impede quality of life [5]. Despite increased understanding of AML biology, minimal progress has been made in the standard induction 7+3 chemotherapy (7 days of cytarabine plus 3 days of an anthracycline, followed by consolidation chemotherapy) and hematopoietic stem cell transplantation over the last four decades [6]. Therefore, novel approaches that are more effective and less toxic are required for a complete cure of AML.

The 90 kDa RSK family (RSK, p90^rsk^) of proteins are expressed in various tissues. RSKs are activated downstream of the Ras-MAPK pathway by ERK1/2 phosphorylation [7]. The ERK phosphorylation site is conserved across the four vertebrate RSK isoforms RSK1 (*RPS6KA1*), RSK2 (*RPS6KA3*), RSK3 (*RPS6KA2*), and RSK4 (*RPS6KA6*). These isoforms share 75–80% sequence homology and two functional kinase domains. The C-terminal domain is phosphorylated by another kinases such as extracellular signal-regulated kinase (ERK), which autophosphorylates the N-terminal kinase domain, enabling the N-terminal domain to phosphorylate canonical RSK targets [7, 8]. RSK activity is involved in numerous signaling pathways of cellular proliferation, survival, and migration through the phosphorylation of a wide range of targets, including tuberous sclerosis complex-1/2 (TSC1/2), membrane-associated tyrosine- and threonine-specific CDC2 inhibitory kinase-1 (MYT1), CDC25, p27^KIP1^, cAMP Responsive Element Binding Protein, Bcl-2-associated death promoter (BAD), and death-associated protein kinase (DAPK) [9–12].

Overexpression or hyper-activation of RSKs has been observed in breast cancer, lung cancer, prostate cancer, multiple myeloma, osteosarcoma, and carcinomas, in which they can increase cell motility and promote invasion and metastasis [9]. RSK1 and RSK2 are the major isoforms in bone marrow [7] and are thought to promote cancer cell growth, survival and proliferation [12, 13]. Inhibition of RSK1 and RSK2 has been reported to inhibit the proliferation of various cancer cells [7, 12]. Recent studies have shown that RSK2 activity is required for FLT3-ITD induced- AML [14] as a novel Pim2 target [15]. RSK2 is also involved in Fibroblast growth factor receptor 3-mediated hematological malignancies in a two-step fashion, promoting both the ERK-RSK2 interaction and subsequent phosphorylation of RSK2 by ERK [16, 17]. RSK-mediated phosphorylation inactivates Myt1 and activates CDC25C, leading to the activation of CDK1 for G2–M cell cycle progression [13, 18].

The spindle assembly checkpoint (SAC), also called the mitotic checkpoint, is a safety surveillance device that monitors proper interactions between kinetochores and spindle microtubules to ensure accurate chromosome segregation into daughter cells. SAC blocks the metaphase-to-anaphase transition until all chromosomes attach to the kinetochores with equal tension across sister kinetochores [19, 20]. The unattached kinetochores initiate the formation of the mitotic checkpoint complex (MCC) composed of MAD2, BUBR1/Mad3, BUB3, and CDC20, leading to the inactivation of CDC20 for proper activity of the anaphase-promoting complex/cyclosome (APC/C). After the kinetochores of all chromosomes are attached to the spindle microtubules in a bidirectional manner, MCC disappears and APC/C is activated for degradation of Cyclin B and Securin, leading to mitotic exit [19–22]. Treatment of cells with microtubule targeting agents (MTAs), including paclitaxel and the vinca alkaloid vincristine, blocks the proper formation of the mitotic spindle through inhibition of microtubule dynamics, resulting in the prolonged mitotic arrest of cancer cells [23]. MTAs-treated mitotic arrested cells may undergo apoptosis in mitosis, however, the rapid degradation of Cyclin B due to an insufficient SAC leads to the mitotic slippage into tetraploid G1 stage in resistant cells [24]. Though vinca alkaloid microtubule-destabilizing compounds have shown clinical efficacy against various hematological malignancies [25] and were included in combination chemotherapy of the VAPA study [26], they are not currently used in induction chemotherapy for AML due to their high toxicity against lymphoid cells and rapid degradation by myeloperoxidase in AML cells [27].

In this study, we demonstrate that BI-D1870, a potent inhibitor of RSK [28], induces mitotic arrest and apoptosis in AML cells without inhibiting CDC2 and CDC25C. Treatment of BI-D1870 inhibited the inactivation of SAC activity, preventing the degradation of Cyclin B and Securin and resulting in blockade of the metaphase/anaphase progression. Furthermore, BI-D1870 synergizes with vinca alkaloid vincristine in AML cells, suggesting that inhibition of mitotic exit with BI-D1870 could be a promising novel approach for AML therapy in combination with MTAs.

## RESULTS

### BI-D1870 induces metaphase arrest in AML cells

**Figure 1:**
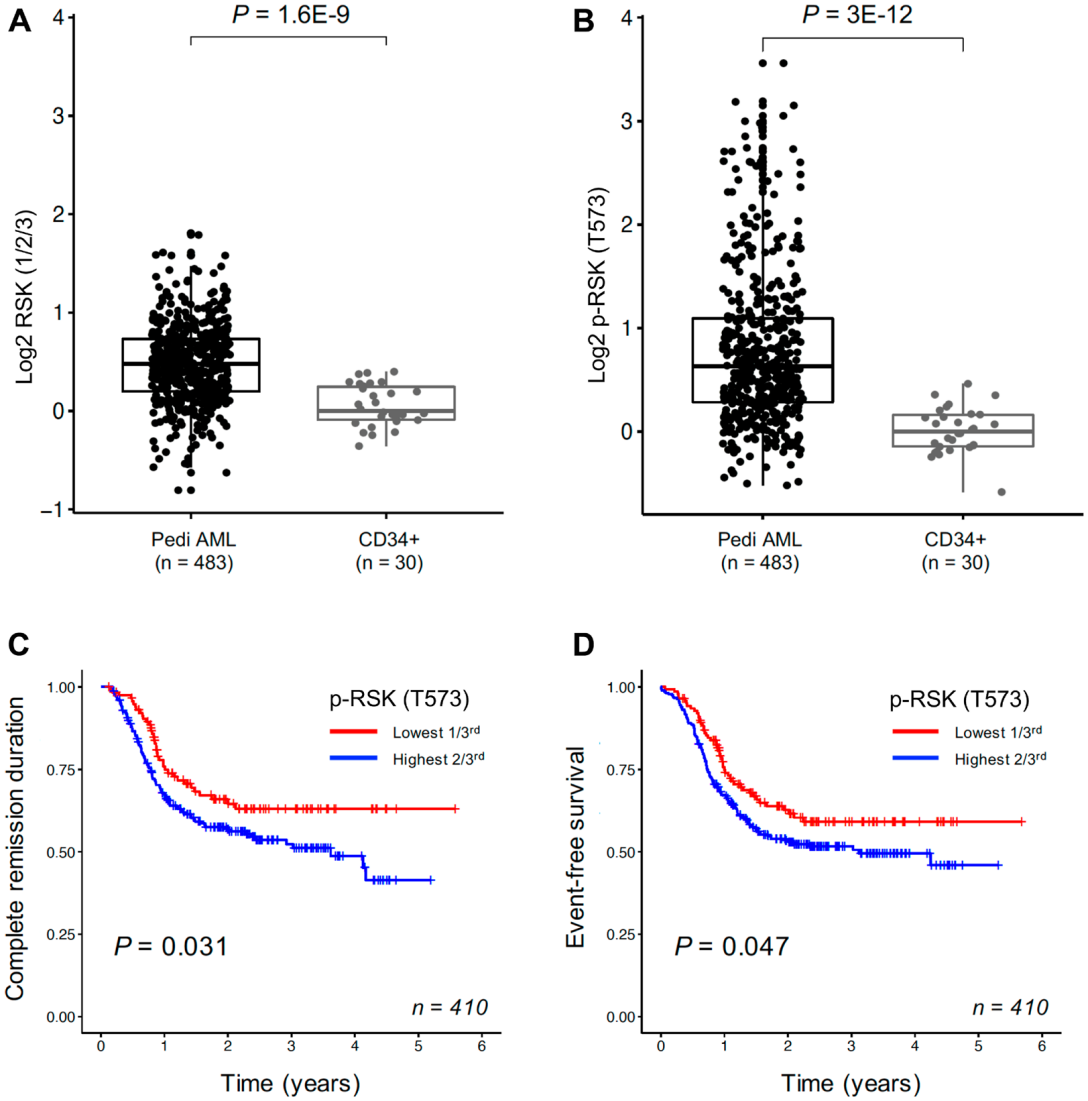
RSKs expression in pediatric AML cells. Reverse phase protein analysis for total RSK (1/2/3) (**A**) and p-RSK (T573) (**B**). Total RSK (1/2/3) protein expression and phosphorylated RSK (T573) in AML blast cells from 483 pediatric patients compared to normal CD34+ samples (10 adults/20 pediatric samples). Both levels of total RSK protein and phosphorylated RSK (T573) were significantly higher in AML cells than normal counter parts. Kaplan–Meier survival curve for complete remission duration and event-free survival in 410 pediatric AML patients. The effect of p-RSK (T573) expression in 410 pediatric AML patients on complete remission duration (**C**) and event-free survival (**D**). Patients were divided into thirds based on their p-RSK (T573) expression, with the lowest third shown in red and the highest two-third in blue. Kaplan–Meier survival curve for event-free survival in 410 pediatric AML patients.

As RSKs have been reported to play a key role in cellular proliferation and survival in cancer cells [9–12], we determined the expression levels of total RSKs (1/2/3) and phosphorylated RSK (T573) proteins in leukemic cells from 483 pediatric AML patients relative to normal CD34+ bone marrow samples (Supplementary Table 1) with Reverse Phase Protein Analysis (RPPA). Phosphorylation at T573 in the C-terminal domain of RSK is important for its activation [7]. Both levels of total RSK (1/2/3) protein and phosphorylated RSK (T573) were significantly higher in pediatric AML leukemic cells compared to normal CD34+ cells (Figure 1A and 1B). Patients with higher levels of phosphorylated RSK (T573) had significantly shorter complete remission duration (*p* = 0.031) and worse event free survival (*p* = 0.047) (Figure 1C and 1D), suggesting hyperactivated RSK could be a drug target for AML therapy. MLL-rearrangement did not affect RSK hyperactivation in AML cells (Supplementary Figure 1).

**Figure 2:**
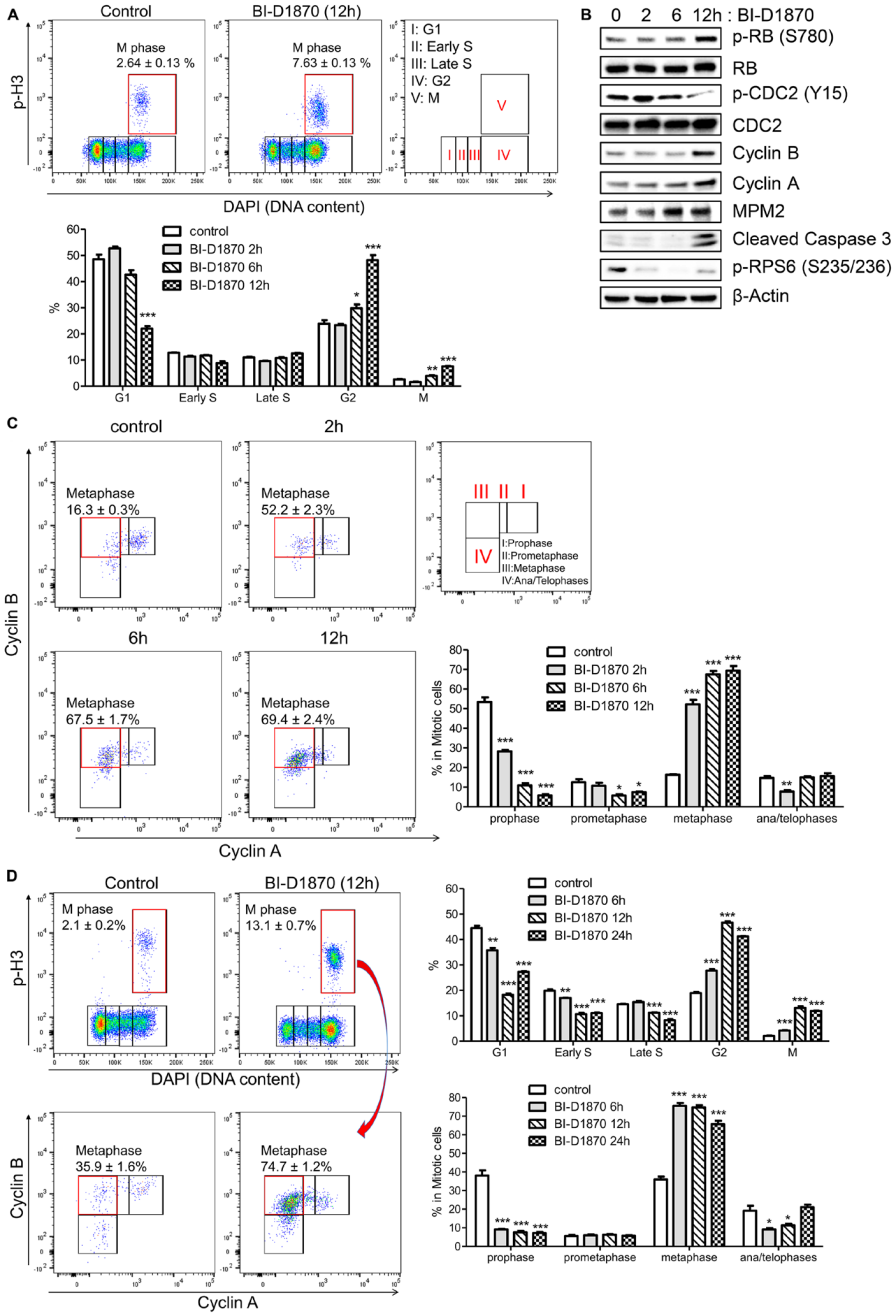
BI-D1870 treatment induces mitotic arrest and apoptosis in HL60 cells. (**A**) Cell cycle profiles of control and BI-D1870-treated HL60 cells by flow cytometry. HL60 cells were treated with BI-D1870 (5 μM). Cells were stained with 4,6-diamidino-2-phenylindole (DAPI) and antibodies. Bivariate distribution of DNA content versus the level of phosphorylated Histone H3 (p-H3) was detected by multi-parameter flow cytometry. Percentages of cell populations at each cell cycle phase determined by DNA content (DAPI) and levels of p-H3, Cyclin A and Cyclin B were graphed. (**B**) Protein levels of G2/M phase markers (p-Rb (S780), p-CDC2 (Y15), Cyclin B and Cyclin A), apoptosis marker (Cleaved Caspase 3) and phosphorylated Ribosomal protein S6 (p-RPS6 (S235/236)) in BI-D1870-treated HL60 cells. Cell extracts were prepared at the indicated times after BI-D1870 treatment. β-Actin was used as an internal control. (**C**) Accumulation of metaphase cells following the treatment of BI-D1870. After treatment with BI-D1870 (5 μM) for the indicated times, HL60 cells were fixed and stained with DAPI and antibodies. Mitotic phases were further characterized in p-H3-positive populations by measuring the levels of Cyclin A and Cyclin B. Data represent the percentages of cell populations residing at each mitotic phase analyzed by the levels of Cyclin A and Cyclin B in the mitotic population. (**D**) Induction of mitotic arrest in KG1 cells by BI-D1870 treatment. KG1 cells were treated with BI-D1870 (5 μM) for indicated times. Cells were fixed and stained with DAPI and antibodies against p-H3, Cyclin A, and Cyclin B. Cell cycle profiles of DMSO or compound-treated KG1 cells were shown as the bivariate distribution of DNA content versus the level of phosphorylated Histone H3 (top). Each mitotic phase distribution was identified as the cellular expression of Cyclin A and Cyclin B in mitotic cells (bottom). The percentage cell population at each cell cycle stage is shown. Flow cytometric profiles represent one out of three experiments with similar results. Data are graphed as mean ± SEM (*n* = 3). ^*^ *p* < 0.05; ^**^ *p* < 0.01; ^***^ *p* < 0.001.

To study the effects of inhibiting RSK in AML, we used a potent RSK inhibitor BI-D1870. We assessed whether RSK inhibition by BI-D1870 decreased viability of AML cell lines. BI-D1870 inhibited cellular viability in a dose-dependent manner with IC_50_ of 1.62, 1.91, and 2.52 μM for MOLM-13, MV-4-11, and HL60 cell lines, respectively (Supplementary Figure 2A), while normal human hematopoietic cells demonstrated no significant decrease in colony formation for up to 10 μM of BI-D1870 (Supplementary Figure 2B). We next examined the effects of BI-D1870 on the cell cycle distribution of HL60 cells. Cell cycle profile was assessed based on the cellular levels of Cyclin A, Cyclin B, mitotic marker phospho-Ser-10 of histone H3 (p-H3), and DNA content. Cyclin A is expressed in S phase cells, maximally expressed in G2/M phase cells, and degraded after post-prometaphase. The cellular level of Cyclin B1 increases at the time of cell exit from S, peaking at mitosis, and decreasing at the onset of anaphase (Supplementary Figure 3) [29–31]. Treatment with BI-D1870 significantly increased cell populations at G2 and M phases (%, control vs. BI-D1870 (5 μM) 12 h, M: 2.6 ± 0.1 vs. 7.6 ± 0.1, G2: 23.9 ± 1.4 vs. 48.2 ± 1.9, mean ± SEM (*n* = 3), *p* < 0.001), and decreased population at G1 phase (%, control vs. BI-D1870 (5 μM) 12 h, 48.5 ± 1.8 vs. 22.0 ± 1.0, mean ± SEM (*n* = 3), *p* < 0.001) (Figure 2A). We next assessed the effect of BI-D1870 on expression of mitotic markers (p-RB (S780), MPM2, and p-CDC2 (Y15)) [32], cyclins, and cleaved Caspase 3, an apoptotic marker, by immunoblotting (Figure 2B). As expected, there was a significant increase in cellular levels of p-RB (S780), MPM2, Cyclin A, and Cyclin B and decrease in p-CDC2 (Y15) following treatment of BI-D1870, showing the accumulation of mitotic cells. BI-D1870 also elicited apoptosis through the activation of Caspase 3 and suppressed the phosphorylation of RPS6 (S235/236), a known direct target of RSK [33]. We evaluated cell cycle progression with BI-D1870 treatment at each mitotic phase. The fraction of cells in prophase, prometaphase, metaphase, and late mitosis can be determined by the expression levels of Cyclin A and Cyclin B in a p-H3-positive population [30, 31]. Metaphase was defined as a p-H3-positive/Cyclin A-negative/Cyclin B-positive population. Surprisingly, the metaphase population was rapidly increased over 3-fold with reduction of the prophase population after 2 h treatment of BI-D1870 (Metaphase (% in mitotic cells), control vs. BI-D1870 (5 μM): 16.3 ± 0.3 vs. 52.2 ± 2.3; Prophase: 53.5 ± 2.3% vs. 28.2 ± 0.8, mean ± SEM, *n* = 3, *p* < 0.01) (Figure 2C), while G2 phase population was increased by 25% after 6 h and 2-fold after 12 h treatment of BI-D1870 (Figure 2A), suggesting that metaphase is a direct target of BI-D1870. BI-D1870 also enriched the cell population at metaphase in another AML cell line, KG1 cells (Figure 2D).

**Figure 3:**
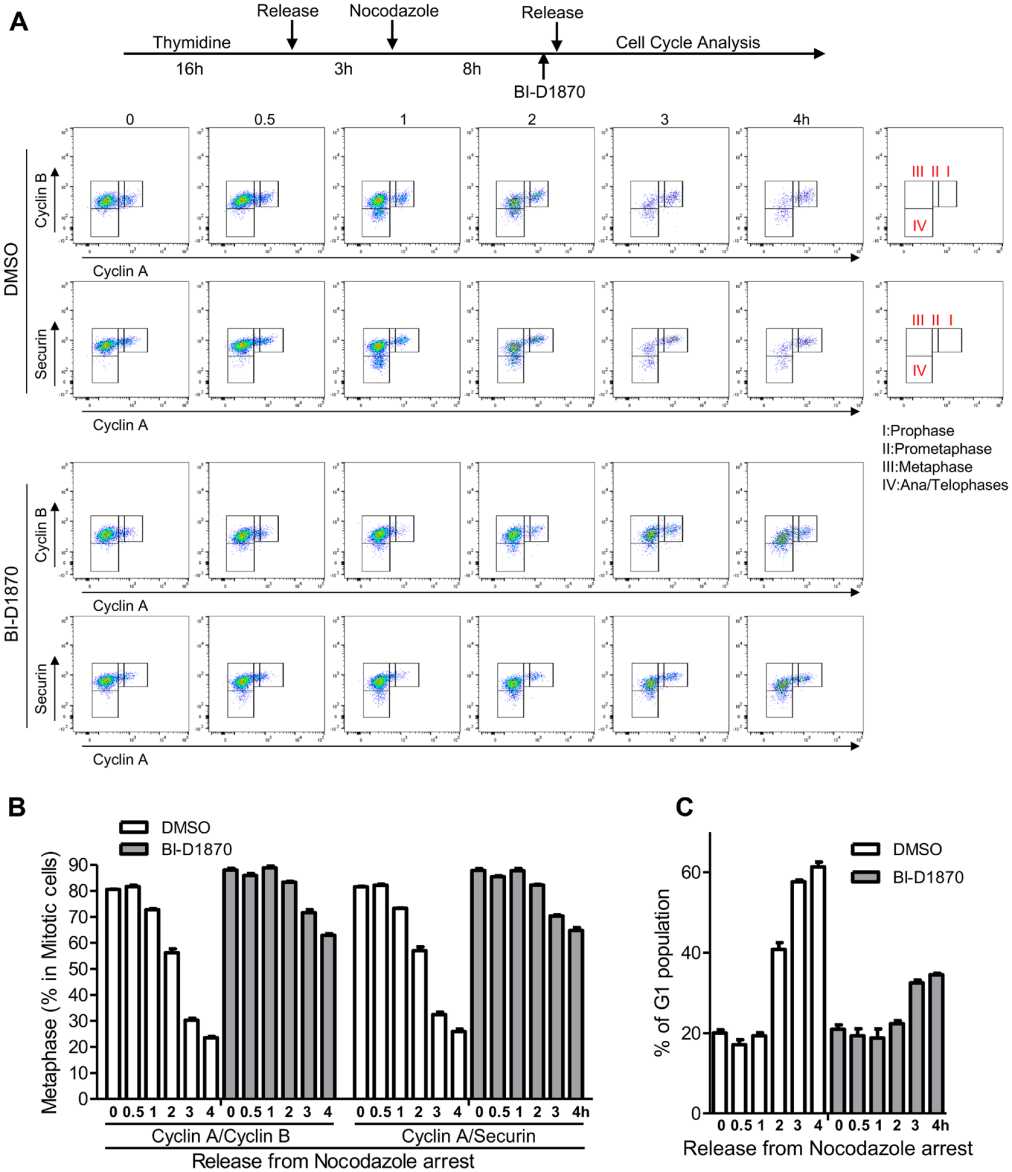
Metaphase arrest by the treatment of BI-D1870. (**A**) HL60 cells were synchronized at metaphase using a thymidine plus nocodazole block. One hour before release, cells were treated with BI-D1870 (5 μM). Cells were released from the nocodazole block (metaphase arrest) and collected at the indicated times with or without BI-D1870 (5 μM). Flow cytometry profiles show the bivariate distribution of expression of Cyclin B versus expression of Cyclin A or Securin in p-H3-positive mitotic cells. The transition from prophase to ana/telophase is characterized by the cellular levels of Cyclin A, Cyclin B, and Securin. Cells started to enter anaphase by 1 h after release. G1 phase population was identified by DNA content and protein levels of p-H3, Cyclin A, and Cyclin B. Plots represent one out of three experiments. Percentages of cell populations residing at metaphase (**B**) or G1 phase (**C**) were calculated using FlowJo software and are expressed as mean ± SEM (*n* = 3).

We then further investigated how cell cycle progression through the mitotic phase was affected by BI-D1870 through the time-lapse measurements of cell population percentage residing at G1, prophase, prometaphase, metaphase, and ana/telophases cells of synchronized HL60 cells following release from the mitotic block. APC/C ubiquitinates and degrades Cyclin A at prometaphase. Cyclin B and Securin are degraded by APC/C at the metaphase-to-anaphase transition, leading to mitotic exit [19–21]. We examined mitotic cell cycle progression by measuring the expression levels of Cyclin A, Cyclin B, and Securin in a p-H3-positive population (Figure 3). Our results show that the metaphase-to-anaphase transition is significantly inhibited by BI-D1870 treatment as assessed by the delayed degradation of Cyclin B and Securin following release from metaphase arrest (metaphase cells in the mitotic population, based on Cyclin A/Cyclin B, %, control vs. BI-D1870, 0 h post-release: 80.5 ± 0.1 vs. 88.0 ± 0.7; 2 h post-release: 56.3 ± 1.4 vs. 83.4 ± 0.3; 4 h post-release: 23.5 ± 0.5 vs. 62.9 ± 0.6, mean ± SEM, *n* = 3, *p* < 0.01). Degradation of Cyclin B and Securin started at 1 h post-release, and mitotic cells entered G1 phase after 2 h post-release in the control group. BI-D1870 significantly inhibited Cyclin B and Securin degradation and mitotic exit to G1 phase, even beyond 4 h post-release from metaphase arrest (G1 phase (%), control vs. BI-D1870, 0 h post-release: 20.0 ± 0.8 vs. 20.9 ± 1.1; 2 h post-release: 40.8 ± 1.7 vs. 22.3 ± 0.8; 4 h post-release: 61.4 ± 1.2 vs. 34.5 ± 0.4, mean ± SEM, *n* = 3).

**Figure 4:**
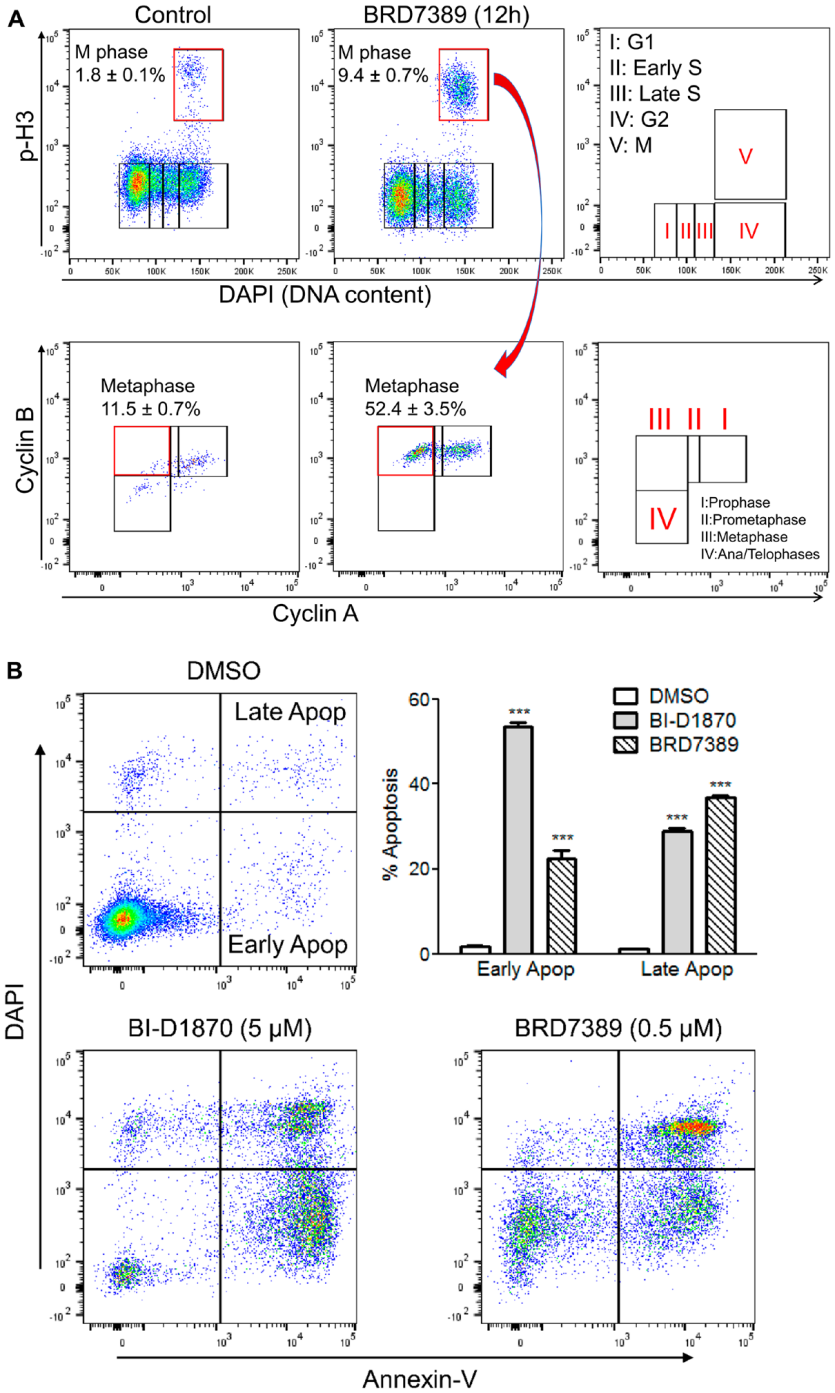
BRD7389 treatment induces metaphase arrest and apoptosis in HL60 cells. (**A**) Bivariate distribution of DNA content versus level of phosphorylated Histone H3 or expression level of Cyclin B versus level of Cyclin A. HL60 cells were treated with BRD7389 (0.5 μM) for 12 h. Metaphase population was determined by DNA content and cellular levels of p-H3, Cyclin A, and Cyclin B. (**B**) BRD7389 induces apoptosis in AML cells. HL60 cells were treated with BRD7389 (0.5 μM) or BI-D1870 (5 μM) for 2d. Apoptosis was assessed by Annexin-V/DAPI staining. Early apoptotic (Annexin-V+/DAPI−) and late apoptotic (Annexin-V+/DAPI+) populations were significantly enhanced following treatment with RSK inhibitors. Flow cytometric profiles represent one out of three independent experiments. Values are indicated as mean ± SEM (*n* = 3). ^***^ *p* < 0.001.

We evaluated the effect of RSK inhibition on metaphase arrest using another RSK inhibitor, BRD7389 [34]. A similar accumulation of cells at metaphase was shown with BRD7389 (Figure 4A). Given RSK plays a key role in cell survival by inhibiting DAPK and BAD [9–12], we assessed the RSK inhibitor-induced apoptosis using annexin V/4′,6-diamidino-2-phenylindole (DAPI) double staining. Clear early apoptosis (Annexin-V+/DAPI-) and late apoptosis (Annexin-V+/DAPI+) were observed in HL60 cells with both BI-D1870 and BRD7389 treatment (Figure 4B). Both BI-D1870 and BRD7389 induced metaphase arrest and apoptosis despite having different structures and specificities, highlighting that RSK inhibition is critical for the anti-leukemic activity of both drugs.

**Figure 5:**
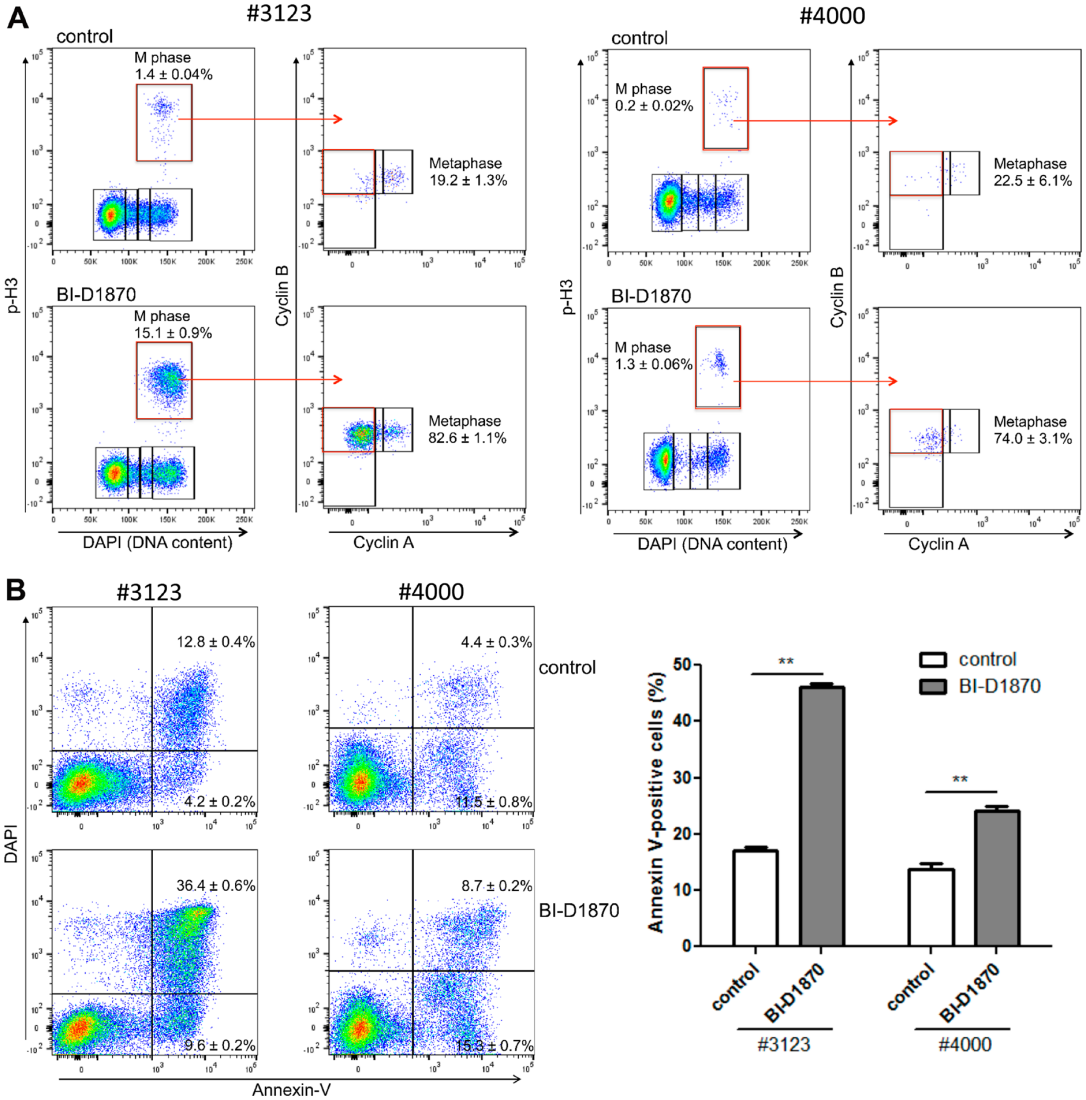
BI-D1870 treatment induces metaphase arrest and apoptosis in primary patient AML cells. (**A**) Bivariate distribution of DNA content versus level of phosphorylated Histone H3 or expression level of Cyclin B versus the level of Cyclin A in mitotic cells. Patient primary AML cells were cultured for 1 day with or without BI-D1870 (5 μM). Cell cycle profile was characterized by DNA content and protein levels of p-H3, Cyclin A, and Cyclin B. Metaphase population was determined by assessing cellular levels of Cyclin A and Cyclin B in p-H3-positive mitotic cells. (**B**) Patient primary AML cells were treated with BI-D1870 (5 μM) for 2d. Apoptotic cells were assessed by Annexin-V/DAPI double staining. Plots are representative of three experiments. Percentages of Annexin V-positive apoptotic cells are shown as mean ± SEM (*n* = 3). ^**^ *p* < 0.01.

We examined the inhibitory effects of BI-D1870 on cell cycle progression and survival in primary AML patient samples. We found that the BI-D1870 induced metaphase population accumulation as well as apoptosis in primary AML cells (Figure 5). BI-D1870 treatment induced higher apoptosis and metaphase population accumulation in #3123 AML cells having more proliferative activity than #4000 cells. Taken together, these data suggest that RSK inhibitors interrupt the metaphase-to-anaphase transition and induce apoptosis in AML cells.

### CDC25C/CDC2 are not targets of BI-D1870 for metaphase arrest

**Figure 6:**
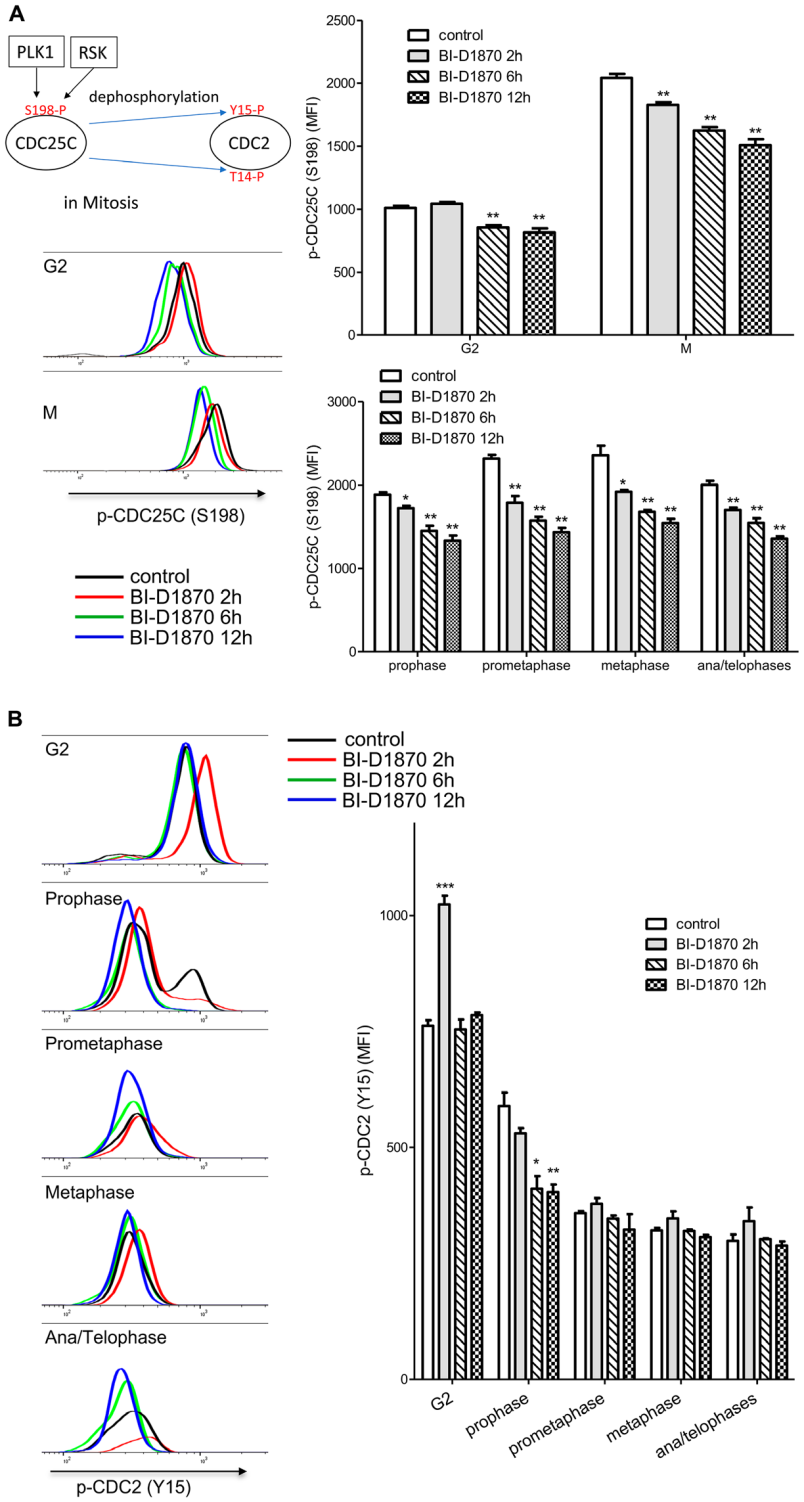
Effect of BI-D1870 on CDC2 and CDC25C activation. HL60 cells were treated with BI-D1870 (5 μM) for the indicated hours, and then cells were collected and analyzed for the cellular levels of p-CDC25C (S198) (**A**) or p-CDC2 (Y15) (**B**). Fixed cells were stained with DAPI and antibodies against Cyclin A, Cyclin B, p-H3, p-CDC2 (Y15), and p-CDC25C (S198). (A) Decrease in positive phosphorylation at serine 198 (S198) on CDC25C following the treatment of BI-D1870. Representative plot of p-CDC25C (S198) levels in G2 and mitotic phases populations following the treatment of BI-D1870. The graph shows the MFI of p-CDC25C (S198) in G2 and mitotic phases populations of HL60 cells treated with or without BI-D1870. (B) Temporary inhibition of CDC2 activation in the G2 phase by BI-D1870 treatment. CDC2 activity is regulated in a negative fashion by phosphorylation at tyrosine 15 (Y15). Representative flow cytometric profile of p-CDC2 (Y15) levels in the G2 and mitotic phases populations following the treatment of BI-D1870. The graph shows median fluorescence intensities (MFI) of p-CDC2 (Y15) in the G2 and mitotic phases populations of HL60 cells treated with or without BI-D1870. Negative phosphorylation at Y15 on CDC2 was transiently enhanced in only the G2 phase at 2 h after BI-D1870 treatment. Flow cytometric profiles represent one out of three independent experiments. Values are graphed as mean ± SEM (*n* = 3). ^*^ *p* < 0.05; ^**^ *p* < 0.01.

RSK has been shown to activate CDC2 for G2/M cell cycle progression by dephosphorylating the negative regulatory phosphorylation at Y15 and T14 residues through activation of CDC25 or inhibition of MYT1 [9, 13, 18]. CDC25C phosphatase activity is critical for the dephosphorylation at T14 and Y15 and subsequent activation of CDC2 during mitosis progression [35]. Phosphorylation at S198 correlates with CDC25C activity and overexpression of RSK increases the cellular level of S198-phosphorylated CDC25C [18]. As expected, phosphorylation at S198 of CDC25C was increased 2-fold when cells entered mitosis, while BI-D1870 decreased S198 phosphorylation by around 20% (Figure 6A). We then examined phosphorylation at Y15 of CDC25, a direct target of CDC25C. The phosphorylation level of CDC2 at Y15 was abruptly decreased upon mitosis entry at prophase, consistent with S198 phosphorylation of CDC25C. While BI-D1870 treatment increased phosphorylation at Y15 of CDC2 transiently in the G2 phase, phosphorylation at Y15 of CDC2 at prophase remained significantly decreased following treatment with BI-D1870, in fact even more so than compared to control (Figure 6B). Though phosphorylation of the positive regulatory S198 of CDC25C was decreased in G2 and mitotic populations, BI-D1870 did not inhibit CDC2 activity in mitosis as measured by durable dephosphorylation at a negative regulatory site, suggesting that inhibition of CDC25C/CDC2 is not a critical regulatory mechanism in BI-D1870-induced mitotic arrest.

### BI-D1870 inhibits dissociation of CDC20 from MAD2

**Figure 7:**
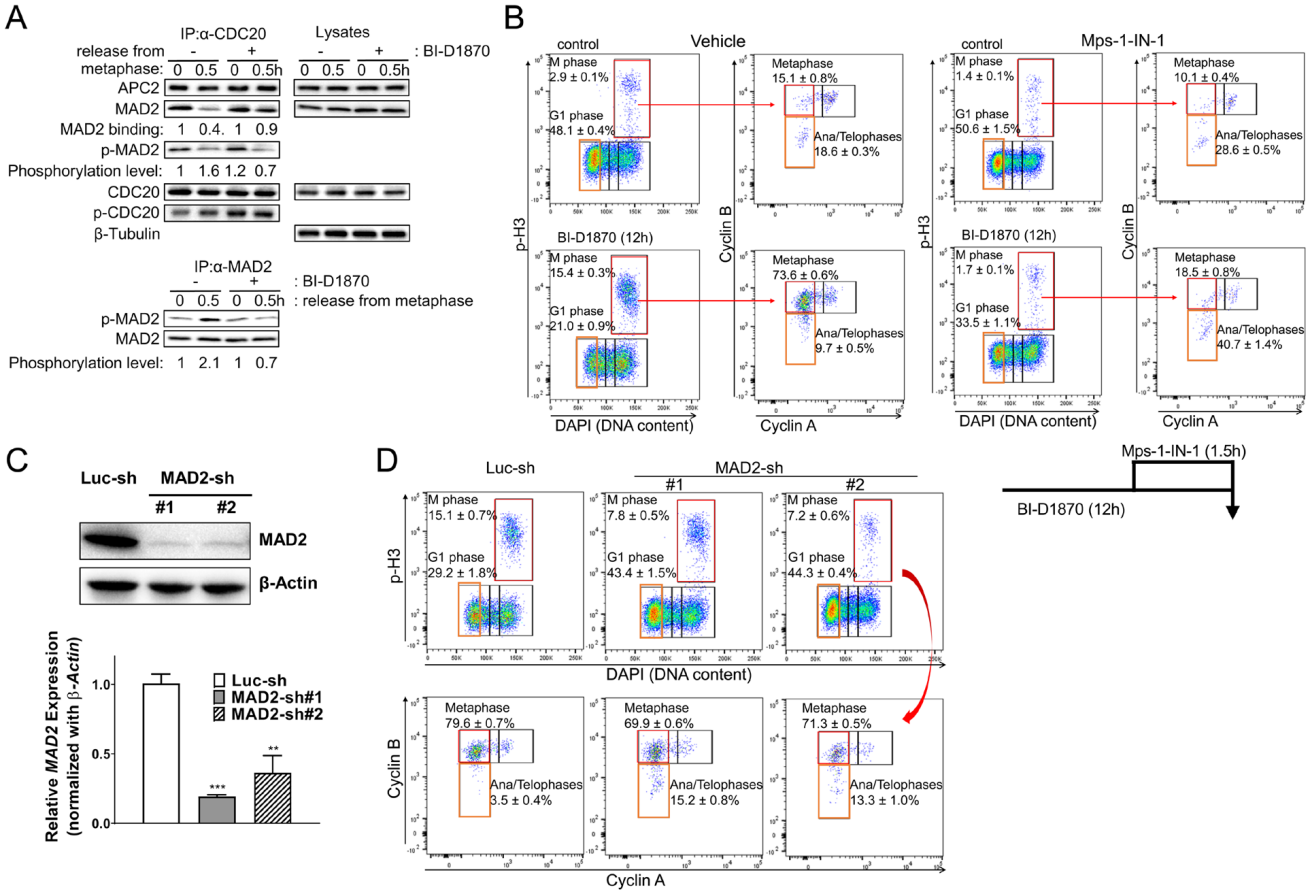
BI-D1870 prolongs the SAC signal. (**A**) BI-D1870 inhibits the association of CDC20 with APC/C complex. HL60 cells were synchronized at metaphase using a thymidine/nocodazole double block. One hour before release, cells were treated with BI-D1870 (5 μM). Cells were released and cultured in media with or without BI-D1870 (5 μM) for 0.5 hours. Total lysates were immunoprecipitated using an anti-CDC20 or anti-MAD2 antibody. IP products and total lysates were analyzed by immunoblotting for APC2, MAD2, phospho-Serine/Threonine, CDC20 and β-Tubulin as a loading control. Relative amounts of bound MAD2 or Ser/Thr-phosphorylated MAD2 were quantified by densitometric measurements. (**B**–**D**) Inactivation of SAC or MAD2 knockdown releases cells from BI-D1870-induced metaphase arrest. (B) Mps1-IN-1 treatment completely released cells from metaphase arrest. HL60 cells were cultured with BI-D1870 (5 μM) or DMSO vehicle control for 12 h. Mps1 kinase inhibitor Mps1-IN-1 (10 μM) was added 90 min before collecting cells to assess the effect of SAC inhibition on BI-D1870-induced metaphase arrest. Cells were fixed and stained with DAPI and antibodies against Cyclin A, Cyclin B, and p-H3. Bivariate flowcytometric profiles show cell populations at each cell cycle stage. (C) MAD2 knockdown alleviates the RSK inhibitor BI-D1870-induced metaphase arrest. HL60 cells were transduced with pLKO.1 lentiviral vector expressing MAD2 shRNA or luciferase shRNA and then selected with puromycin. Suppressed expression of MAD2 was confirmed at the protein level by immunoblotting and in mRNA levels by qRT-PCR. (D) MAD2 knockdown cells were treated with BI-D1870 (5 μM) for 12 hours, and then cells were fixed and analyzed for cell cycle distribution. Fixed cells were stained with DAPI and antibodies against Cyclin A, Cyclin B, and p-H3. The mitotic cell population was determined by DNA content and protein levels of p-H3, Cyclin A, and Cyclin B (top). Flow cytometric profiles represent one out of three experiments with similar results.

As BI-D1870 did not stabilize Cyclin A in mitosis (Figure 3), the activity of APC/C with coactivator CDC 20 itself was not inhibited by BI-D1870. Cyclin B and Securin are protected from APC/C by the MCC that is activated by SAC and MTAs [22, 36]. Therefore, we hypothesized that BI-D1870 could induce a chronic generation of MCC complex in the absence of spindle damage, resulting in a prolonged metaphase arrest. We examined whether BI-D1870 affects the formation of the MCC using a co-immunoprecipitation experiment. While CDC20 co-immunoprecipitated with both APC2 and MAD2 in metaphase and MAD2 dissociated from CDC20 after release from metaphase arrest, BI-D1870 maintained the MAD2/CDC20 complex even after release (Figure 7A). C-terminal residues (S170, S178, S185, and S195) of MAD2 are phosphorylated, and MAD2 phosphorylation plays a critical role in the inactivation of SAC for anaphase progression [37]. BI-D1870 inhibited the phosphorylation of MAD2 at anaphase transition (Figure 7A).

If BI-D1870 inhibits SAC inactivation, the ablation of SAC function should relieve the BI-D1870-induced metaphase arrest. Mps1 phosphorylates Knl1 to recruit SAC proteins, including MAD2, BUBR1/Mad3, BUB3, and CCD20 for MCC assembly [22]. Therefore, we investigated whether Mps1 inhibition could abrogate the BI-D1870 effect in mitosis. Inactivation of SAC with the Mps1inhibitor Mps1-IN-1, which blocks the activation of SAC [38], increased the population of ana/telophases and decreased the metaphase population (Figure 7B and Supplementary Figure 4A). Moreover, Mps1 inhibition removed BI-D1870-induced metaphase arrest in 90 minutes. Mps1-IN-1 treatment enhanced the populations at G1 and ana/telophases (control vs. Mps1-IN-1: G1 (%), 21.0 ± 0.9 vs. 33.5 ± 1.1; ana/telophases in M (%), 9.7 ± 0.5 vs. 40.7 ± 1.4. mean ± SEM, *n* = 3, *p* < 0.001) and decreased the populations at total mitotic phase and metaphase (control vs. Mps1-IN-1: M (%), 15.4 ± 0.3 vs. 1.7 ± 0.1; metaphase in M (%), 73.6 ± 0.6 vs. 9.7 ± 0.8. mean ± SEM, *n* = 3, *p* < 0.001) (Figure 7B and Supplementary Figure 4A). To further examine whether SAC was activated by BI-D1870, we analyzed the effect of BI-D1870 on cell cycle progression in MAD2 knockdown cells. MAD2 knockdown efficiency was assessed by immunoblotting and qRT-PCR (Figure 7C). As shown earlier, following BI-D1870 treatment, HL60 cells were accumulated at metaphase after 12 h. In contrast, MAD2 knockdown HL60 cells were resistant to BI-D1870 treatment with prominent increase in G1 and ana/telophases populations (control vs. MAD2-knockdown #1 vs. MAD2 knockdown #2: G1 (%), 29.2 ± 1.8 vs. 43.4 ± 1.5 vs. 44.3 ± 0.4; ana/telophases in M (%), 3.5 ± 0.4 vs. 15.2 ± 0.8 vs. 13.3 ± 1.0. mean ± SEM, *n* = 3, *p* < 0.01), in concurrence with the suppression of mitotic cell population (control vs. MAD2-knockdown #1 vs. MAD2 knockdown #2: M (%), 15.1 ± 0.7 vs. 7.8 ± 0.5 vs. 7.2 ± 0.6; metaphase in M (%), 79.6 ± 0.7 vs. 69.9 ± 0.6 vs. 71.3 ± 0.5. mean ± SEM, *n* = 3, *p* < 0.01) (Figure 7D and Supplementary Figure 4B). MAD2 knockdown also abrogated the BI-D1870-induced effects on mitosis in another AML cell line KG1 (Supplementary Figure 5). These data indicate that sustained activation of SAC is a major mechanism by which BI-D1870 induces metaphase arrest.

### BI-D1870 sensitizes AML cells to vincristine

**Figure 8:**
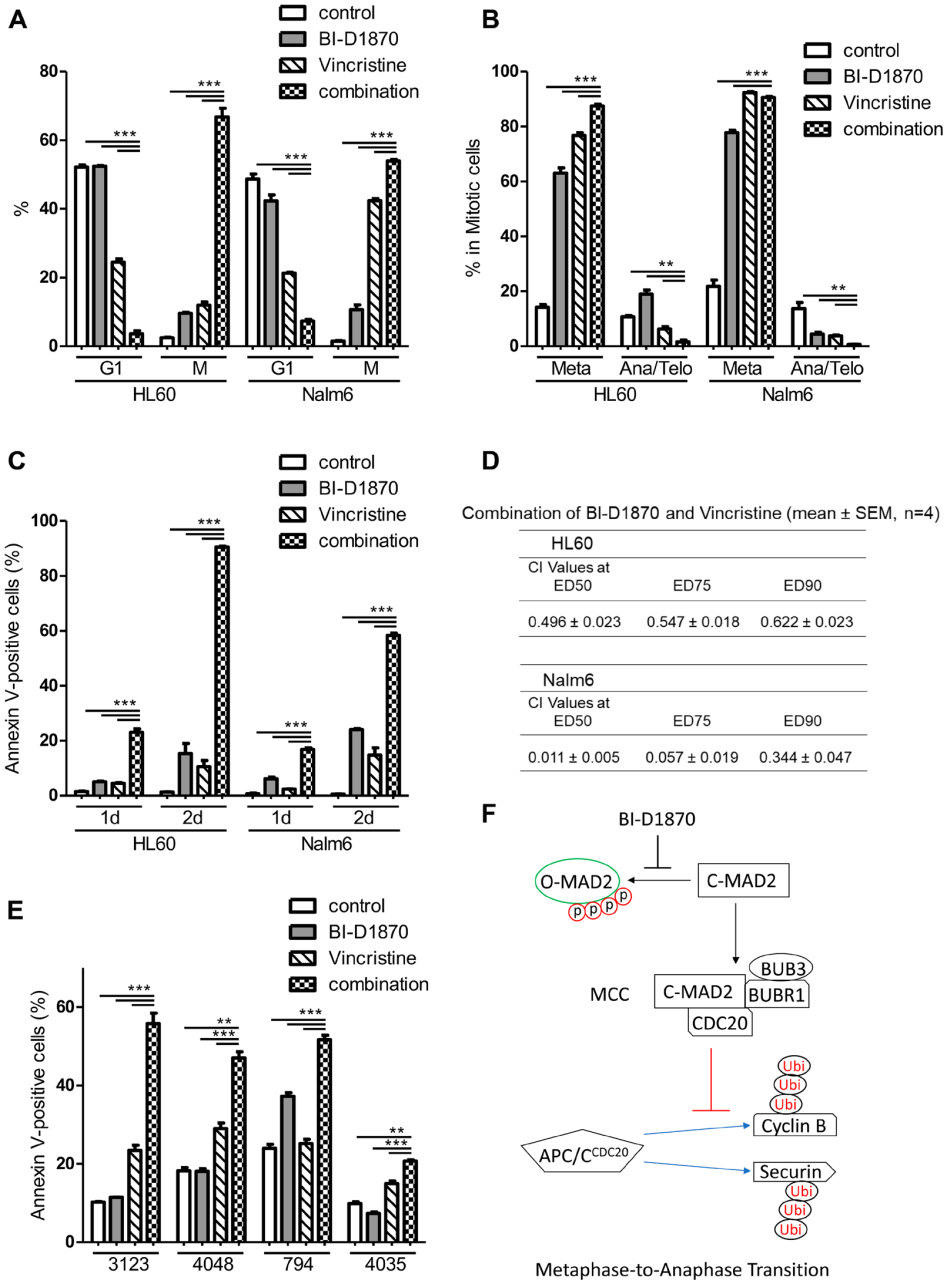
BI-D1870 in combination with vincristine increase metaphase arrest and apoptosis synergistically. (**A**, **B**) Cell cycle distribution of HL60 and Nalm6 cells treated with BI-D1870 and vincristine. Cells were treated with BI-D1870 (2.5 μM) and/or vincristine (5 nM) for 1 d, and then cells were fixed and analyzed for cell cycle distribution. Cells were stained with 4,6-diamidino-2-phenylindole (DAPI) and antibodies. Percentages of live cell populations at each cell cycle phase determined by DNA content (DAPI) and levels of p-H3, Cyclin A, and Cyclin B were graphed. (**C**) Cells were treated with BI-D1870 (2.5 μM) and/or vincristine (5 nM) for 1d or 2d. Percentages of Annexin V-positive apoptotic cells are calculated. (**D**) Synergistic combination effect of BI-D1870 and vincristine. Cells were treated with various concentrations of BI-D1870 and vincristine for 3d. Viability was assessed using the CellTiter glo assay kit. The combination index (CI) values were calculated by the Chou-Talalay method using CalcuSyn software. CI > 1: antagonism, CI = 1: additive Effect, CI < 1: synergism. Data are the mean ± SEM (*n* = 4). (**E**) BI-D1870 in combination with vincristine-induced apoptosis synergistically in primary patient AML cells. Primary cells were treated with BI-D1870 (1.5 μM) and/or vincristine (3 nM) for 2d, and then Annexin V-positive cells were assessed using flow cytometry. Data are graphed as mean ± SEM (*n* = 3). ^**^ *p* < 0.01; ^***^ *p* < 0.001. (**F**) A proposed action model of the RSK inhibitor BI-D1870 in mitotic exit. BI-D1870 inhibits the disassembly of the MCC to activate APC/C for anaphase transition by blocking the phosphorylation-induced conformation change of MAD2.

Microtubule targeting antimitotic drugs including paclitaxel and vincristine are amongst the most successful chemotherapeutics [23, 39]. However, cancer cells can escape from cell death by antimitotic drugs through mitotic slippage [40]. Vincristine is not usually used in current AML treatment due to the rapid inactivation in AML cells [27]. Therefore, we examined whether BI-D1870 can sensitize AML cells to vincristine. We used vincristine and BI-D1870 at concentrations low enough to cause submaximal mitotic arrest and apoptosis in HL60 cells. Either vincristine (5 nM) or BI-D1870 (2.5 μM) alone increased M phase population up to10% of the cell cycle. Treatment with both compounds in combination increased the mitotic population and decreased G1 population synergistically in both cells (HL60 cells, control vs. BI-D1870 vs. vincristine vs. combination: G1 (%), 52.1 ± 0.7 vs. 52.4 ± 0.3 vs. 24.5 ± 2.8 vs. 3.6 ± 0.9; M (%), 2.5 ± 0.2 vs. 9.6 ± 0.2 vs. 12.0 ± 0.9 vs. 66.8 ± 2.5. mean ± SEM, *n* = 3) (Figure 8A). While vincristine alone induced huge mitotic arrest in acute lymphoid leukemia cell line Nalm6, HL60 cells were resistant to vincristine (mitotic cells after 1d treatment of vincristine (%), Nalm6 vs. HL60: 42.4 ± 0.7 vs. 12.0 ± 0.9. mean ± SEM, *n* = 3, *p* < 0.001). However, combination treatment of vincristine and BI-D1870 showed higher accumulation of mitotic population in HL60 cells than Nalm6 cells (mitotic cells after 1d combination treatment (%), Nalm6 vs. HL60: 54.0 ± 0.4 vs. 66.8 ± 2.5. mean ± SEM, *n* = 3, *p* < 0.01) (Figure 8A). Combination treatment also enhanced metaphase accumulation in mitotic cells compared with a single treatment in HL60 cells (control vs. BI-D1870 vs. vincristine vs. combination: metaphase in M (%), 14.1 ± 1.1 vs. 63.0 ± 1.9 vs. 76.7 ± 1.1 vs. 87.4 ± 0.6. mean ± SEM, *n* = 3) (Figure 8B).

To investigate whether BI-D1870-induced sensitization of cells to vincristine by causing metaphase arrest synergistically enhances the cell death in AML cells, we examined the apoptosis of vincristine-treated cells with or without BI-D1870. Each compound alone had minimal effect on inducing apoptosis in both HL60 and Nalm6 cells. The combination treatment of vincristine and BI-D1870 significantly induced apoptosis compared to control and single treatment groups. Consistent with the degree of metaphase arrest, combination treatment induced higher apoptosis in HL60 cells than Nalm6 cells (Annexin V-positive cells (%, 2d combination treatment), Nalm6 vs. HL60: 58.4 ± 0.8 vs. 90.8 ± 0.3. mean ± SEM, *n* = 3, *p* < 0.001) (Figure 8C). While 1 day of treatment with the combination of vincristine and BI-D1870 was sufficient to induce metaphase arrest, subsequent apoptosis was significantly shown 2 days after treatment (Figure 8A and 8C), indicating that leukemic cells undergo apoptosis after the mitotic block following combination treatment of vincristine and BI-D1870. To confirm the synergistic effects of the combination of vincristine and BI-D1870 on leukemic cell viability, HL60 and Nalm6 cells were treated with both drugs at a fixed ratio of IC_50_ for vincristine and BI-D1870 to obtain combination indices (CI). The combination of vincristine and BI-D1870 showed a synergistic combination effect with CI values of < 1 in both cell lines (Figure 8D). We also performed combination experiments to assess the efficacy of combining BI-D1870 with cytarabine or daunorubicin, standard chemotherapeutic agents in current AML therapy. A combination of BI-D1870 and cytarabine or daunorubicin in HL60 cells showed an additive effect against HL60 cell viability (Supplementary Table 2). We further tested the combination of vincristine and BI-D1870 in primary patient AML cells. We cultured four different primary AML cells with cytokines for 2 days with compounds. Combination treatment of vincristine (3 nM) and BI-D1870 (1.5 μM) led to a significantly higher increase in apoptosis than single treatments (Figure 8E).

## DISCUSSION

Hyperactivation of RSKs is a critical factor in survival, proliferation, and metastasis in several types of cancer cells [9–12]. In this study, we demonstrated that RSK is overexpressed and hyperactivated in AML, and that high levels confer an adverse prognosis, suggesting RSK inhibition as a potential therapeutic target in AML. We have shown that the RSK inhibitor BI-D1870 impaired metaphase-to-anaphase transition and induced apoptosis. Moreover, BI-D1870-induced blockade of mitotic exit sensitized AML cells to vincristine.

CDC2 is phosphorylated at Y15 by WEE1 kinase and at T14 by MYT1, which keeps CDC2 inactive before mitosis [41]. CDC25C phosphatase activity is critical for the dephosphorylation at T14 and Y15 and subsequent activation of CDC2 during mitosis progression [35]. RSK is involved in the activation of CDC2 for G2/M cell cycle progression by dephosphorylating at Y15 and T14 by activating CDC25C [9, 13, 18]. CDC25C phosphorylation at S198 enhances its phosphatase activity and nuclear localization in prophase [18]. Though phosphorylation at Ser198 was suppressed by BI-D1870 during mitosis, phosphorylation level at Y15 of CDC2 was not increased in mitosis following treatment of BI-D1870, suggesting that inhibition of CDC25C/CDC2 is not a critical regulatory mechanism in BI-D1870-induced mitotic arrest.

APC/C is activated in mitosis and degrades Cyclin A at prometaphase when SAC is still turned on. However, APC/C with coactivator CDC20 can ubiquitinate Securin and Cyclin B for the anaphase transition only after SAC is turned off and MCC containing MAD2 disappears [22, 36]. SAC blocks the activation of APC/C toward these substrates that are critical for anaphase transition to ensure proper chromosome segregation. While Cyclin A was degraded in mitosis in the presence of BI-D1870, BI-D1870 treatment stabilized the SAC-inhibitable D-box dependent APC/C substrates, Cyclin B and Securin. Furthermore, inactivating the SAC with Mps1 kinase inhibitor Mps1-IN-1 and MAD2 knockdown relieved BI-D1870-induced metaphase arrest, supporting the idea that BI-D1870 targets the SAC silencing machinery rather than APC/C directly to sustain mitotic arrest. Furthermore, RSK inhibition prevented MAD2 dissociation from CDC20 and MAD2 phosphorylation during anaphase transition. MAD2 phosphorylation at C-terminal residues is involved in the silencing of SAC by blocking the conformational activation of MAD2 [42], implying that RSK activity may contribute to turning SAC off by modulating MAD2 phosphorylation (Figure 8F).

MTAs kill cancer cells following mitotic arrest through the activation of SAC [23]. BI-D1870 potentiates vincristine-induced prolonged mitotic arrest. BI-D1870 may leads to cell death via mitotic catastrophe [43, 44]. As SAC-compromised cancer cells can escape from MTA-induced mitotic arrest before enough apoptotic machinery is activated to induce cell death, targeting mitotic exit is one of the emerging strategies to overcome MTA-resistance in cancer cells [45]. CDC20 ablation induces complete metaphase arrest and cell death in cancer cells [46]. Deregulated expression of mitotic regulators has been found in AML cells, including downregulation of BUB1 and overexpression of Aurora-A, Aurora-B, PLK1, and CDC20, which causes genetic instability and confers resistance to MTAs by inactivating SAC [24, 47, 48]. Therefore, strengthening SAC activity should be a promising strategy to eradicate AML cells by inducing synthetic lethality with MTAs. BI-D1870 significantly enhanced the efficacy of vincristine in inducing metaphase-arrest and subsequent apoptosis, highlighting the importance of inhibition of SAC silencing machinery to treat AML.

BI-D1870 is a reversible pan-RSK inhibitor, showing > 500-fold higher activity for RSK than other AGC kinases [28]. BI-D1870 also inhibits the activity of PLK1, Aurora-B, MELK, PIM3, MST2, and GSK3β [28, 49] at higher concentrations than for RSK. As PLK1 and Aurora-B are required for the maintenance of SAC signaling [50], inhibition of these kinases should relieve cells from mitotic arrest, not prolonging mitotic arrest. BI-D1870 and BRD7389 have been reported to inhibit proliferation and significantly increase the G2/M population in melanoma cells [51]. BI-D1870 does not have proper physicochemical properties for clinical application [52]. Future structure–activity relationships study for BI-D1870 is required to improve solubility and pharmacokinetic profiles for *in vivo* preclinical and clinical studies.

## MATERIALS AND METHODS

### Cell culture

KG1, HL60, and Nalm6 human acute leukemia cells were cultured at 37°C with 5% CO_2_ in Iscove’s Modified Dulbecco’s Medium (IMDM, Life Technologies, Grand Island, NY, USA) supplemented with 10% fetal bovine serum plus 1% penicillin/streptomycin/L-glutamine. Cell lines were obtained from ATCC (Manassas, VA, USA). Low-passage cell stocks were used and cultured for less than 2 months. Cells were regularly tested for Mycoplasma and growth characteristics. For analysis of metaphase-to-anaphase transition, HL-60 cells were synchronized at metaphase using a modified thymidine plus nocodazole block [53]. HL-60 cells were treated with 1.5 mM thymidine (Sigma, St. Louis, MO, USA) for 16 h, washed with PBS and released in fresh media for 3 h. The cells were then treated with 150 nM nocodazole (Sigma) for 8 h. The metaphase synchronized cells were washed with PBS and released from the mitotic block by the addition of normal serum-containing media. BI-D1870 (5 μM, Selleckchem, Houston, TX, USA) or vehicle (DMSO) was added 30 min before mitotic release. For cell viability assays, cells were seeded at 2 × 10^4^ cells/well in 96-well plates. CellTiter-Glo assays (Promega, Madison, WI, USA) were performed to assess the viability of cells. Combination indices were calculated with the Chou-Talalay method using CalcuSyn software (Biosoft, Ferguson, MO, USA) [54]. CI values were used to determine synergism (CI < 1), antagonism (CI > 1) and additivity (CI = 1). Cells were treated with combined drugs at a fixed ratio of IC_50_ for two drugs.

Primary patient AML cells were cultured in IMDM/15% BIT (bovine serum albumin, insulin, transferrin; Stem Cell Technologies, Vancouver, BC, Canada)/SCF (100 ng/ml, Miltenyi Biotec, Auburn, CA, USA)/FLT3-ligand (50 ng/ml, Miltenyi Biotec)/IL3 (20 ng/ml, Miltenyi Biotec)/IL6 (20 ng/ml, Miltenyi Biotec)/TPO (50 ng/ml, Miltenyi Biotec)/GM-CSF (20 ng/ml, Miltenyi Biotec)/55 μM β-mercaptoethanol/0.75 μM SR1 (Selleck Chemicals, Houston, TX, USA)/35 nM UM171 (Stem Cell Technologies) [55, 56]. Bone marrows from AML patients were collected through voluntary patient participation at the University of California, Los Angeles (Los Angeles, CA, USA) and Stanford University (Palo Alto, CA, USA) in compliance with the Institutional Review Board regulations of each institution. Informed consent was obtained from all human subjects, and all research was conducted in accordance with the statements outlined in the declaration of Helsinki and the Data Protection Directive.

### RPPA

The methodology and validation of the RPPA technique are fully described elsewhere [57, 58]. Briefly, fresh samples were isolated and enriched for leukemic cells by CD3/CD19 depletion (Miltenyi Biotech, Cologne, Germany). Protein preparations were prepared and normalized to a concentration of 1 × 10^7^ cells/mL and printed in five serial dilutions onto slides along with normalization and expression controls. Slides were probed with a strictly validated primary antibody against total RSK1/2/3 (Cell Signaling #9347) or p-RSK1/2/3 (T573) (Cell Signaling #9346) and a secondary antibody to amplify the signal, and finally a stable dye was precipitated. Stained slides were analyzed using Microvigene^®^ Software (version 3.0, Vigene Tech, Carlisle, MA, USA) to produce quantified data. *SuperCurve* algorithms were used to generate a single value from the five serial dilutions [59]. Loading control [60] and topographical normalization [61] procedures were performed to account for protein concentration and background staining variations on each array. Peripheral blood samples were obtained from 483 de novo pediatric AML patients that participated in the COG AAML1031 Phase 3 clinical trial, and CD34+ bone marrow samples obtained from 30 healthy donors; 20 pediatric CD34+ samples and 10 adults CD34+ samples. Outcome data was available for 410 of the patients enrolled on the AAML1031 study. One hundred and sixty-four patients received standard ADE induction therapy, 210 patients received ADE in combination with BTZ (ADEB), and 36 received ADE + sorafenib (ADES). No difference in effect of RSK1/2/3 or p-RSK (T573) was observed between the treatment arms.

### MAD2 knockdown

Lentiviral vectors expressing MAD2 (#1: TRCN0000006566, #2: TRCN0000273382) and Luciferase shRNA (SHC007) were purchased from Sigma-Aldrich. Lentiviral particles were prepared by transient calcium phosphate transfection of HEK293 cells. Transduced cells were selected by treating cells with puromycin (Sigma-Aldrich) at 2 μg/mL for 3 days. The efficacy of knockdown of endogenous MAD2 expression was assessed by qRT-PCR and western blot analysis.

### Flow cytometry analysis

For assessing intracellular proteins by flow cytometry, cells were fixed with 1.6% paraformaldehyde for 10 min. Fixed cells were made permeable with BD Perm/Wash buffer (BD Biosciences, San Jose, CA, USA) and incubated with antibodies and DAPI (0.1 ug/ml, Sigma, St. Louis, MO, USA) in BD Perm/Wash buffer. The following antibodies were used in intracellular protein staining: FITC-conjugated anti-Cyclin A (clone BF683), PE-conjugated anti-Cyclin B (clone GNS-1), Alexa Fluor 647 or Alexa Fluor 700-conjugated anti-cPARP (clone F21-852, BD Biosciences); PECy7-conjugated ant-pH3 (pS28) (clone HTA283, Biolegend); anti-Securin (EPR3240, Abcam, Cambridge, MA); anti-p-CDC2 (Y15) (clone 10A11, Cell Signaling Technology, Danvers, MA, USA); Alexa Fluor 647-conjugated goat anti-rabbit IgG (Invitrogen, Carlsbad, CA). Apoptosis was measured by Annexin-V (Biolegend, San Diego, CA, USA)/DAPI double staining. Stained cells were analyzed on a DxP10 FACScan (BD Biosciences/Cytek Development, Fremont, CA, USA) using the FlowJo software (TreeStar, Ashland, OR, USA).

### Immunoprecipitation and immunoblotting

Cells were lysed in cell lysis buffer (50 mM Tris-HCl, pH7.5, 120 mM NaCl, 1 mM EDTA, and 1% NP-40) containing protease inhibitor cocktail (Roche, Indianapolis, IN, USA) and phosphatase inhibitor cocktail 2 (Sigma). Cellular lysates (1 mg) were immunoprecipitated, using the anti-CDC20 (5 μl, AR12, Millipore, Burlington, MA, USA) or anti-MAD2 (5 μg, A300, Bethyl Lab, Montgomery, TX, USA) antibodies. The immunoprecipitated products or total lysates were resolved on SDS polyacrylamide gel electrophoresis and transferred to PVDF membranes (BioRad). Proteins were detected with specific antibodies. The following antibodies were used in western blot analyses: anti-Cyclin B1 (H433), anti- CDC2 [54], anti-β-Tubulin (H-235, Santa Cruz Biotechnology, Santa Cruz, CA, USA); ant-MAD2 [48], anti-phosphoserine/threonine (22A, BD Biosciences); anti-β-Actin (M2, Sigma); anti-cyclin A2 (BF683), anti-cleaved Caspase 3 (D175), anti-p-RPS6 (S235/236) (2211), anti-p-CDC2 (Y15), anti-RB (4H1), anti-p-RB (S780) (D59B7), anti-APC2 (12301, Cell Signaling Technology, Danvers, MA, USA); anti-MPM2 (Millipore); anti-CDC20 (A301, Bethyl Lab). Horseradish peroxidase-conjugated anti-mouse or anti-rabbit IgG antibodies (Cell Signaling Technology; GeneTex, Irvine, CA, USA) were used and proteins were visualized with an enhanced chemiluminescence system (Advansta, Menlo Park, CA, USA).

### RNA extraction and quantitative reverse transcription PCR (qRT- PCR)

Total RNA was extracted from cells using Aurum total RNA mini kit (BioRad, Hercules, CA, USA) according to the manufacturer’s instructions and reverse transcribed into cDNA using iScript cDNA Synthesis Kit (BioRad). PCR was carried out on a CFX384 Real-time PCR system (BioRad) using IQ™ SYBR^®^ Green Supermix (BioRad). β-actin was used as a control gene. Expression levels of target mRNAs were calculated by the 2^−ΔΔCT^ Livak method [62]. Primer sequences (5′ to 3′); *ACTB* F: GGACTTCGAGCAAGAGATGG, *ACTB* R: AGCACTGTGTTGGCGTACAG; *MAD2* F: ATCGTGGCCGAGTTCTTCTC, *MAD2* R: TACAAGCAAGGTGAGTCCGT.

### Statistical analysis

Mean protein expression levels measured in the 483 pediatric patient samples were compared to the mean expression levels of the 30 normal CD34+ cells using the Wilcoxon signed-rank test. Survival curves were plotted using the Kaplan–Meier method. Patients were divided into thirds based on the relative RSK1/2/3-total and RSK1-pT537 expression. The statistical tests and plots were generated in R (Version 0.99.484 –2009-2015 RStudio, Inc.). All other data was analyzed using Prism software (Graphpad, La Jolla, CA, USA) with a *p*-value less than 0.05 considered as a significant difference. For combination treatment analysis, combination index values were calculated by Chou-Talalay method using the CalcuSyn software (Biosoft, Ferguson, MO, USA) as described [54].

## SUPPLEMENTARY MATERIALS


