# Supplementary material for: RSK inhibitor BI-D1870 inhibits acute myeloid leukemia cell proliferation by targeting mitotic exit
Source: Oncotarget. 2020 Jun 23;11(25):2387–403. doi: 10.18632/oncotarget.27630 (PMC7321696; doi:10.18632/oncotarget.27630)
Supplement: Supplementary file 1 [file oncotarget-11-2387-s001.pdf]

RSK inhibitor BI-D1870 inhibits acute myeloid leukemia cell proliferation by targeting mitotic exit

SUPPLEMENTARY MATERIALS

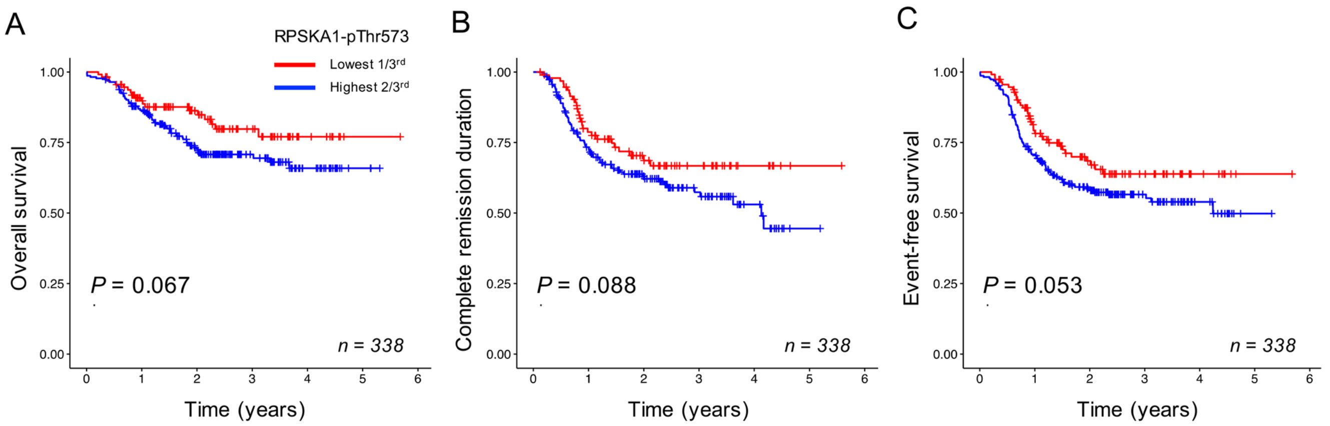

Supplementary Figure 1: Kaplan–Meier survival curve for overall survival, complete remission duration and event-free survival in 338 non-MLL-rearrangement pediatric AML patients. The effect of RPSKA1-pThr573 expression in 338 pediatric AML patients (excluding MLL-rearrangement) on (**A**) overall survival, (**B**) complete remission duration and (**C**) event-free survival. Patients were divided into thirds based on their RPSKA1-pThr573 expression, with the lowest third shown in red and the highest two-third in blue.

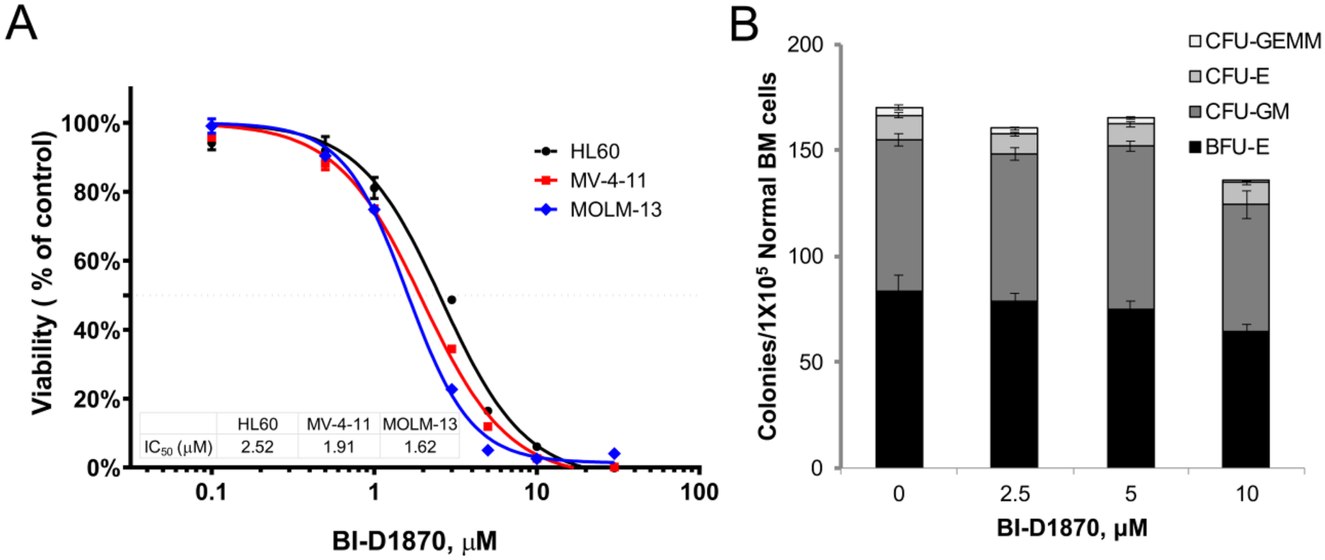

Supplementary Figure 2: Effect of BI-D1870 on AML cell viability and colony forming activity of normal BM cells. (**A**) Dose-response curves are drawn for the AML cell lines HL60, MV-4-11, and MOLM-13 following treated with BI-D1870 for 48 h. Cell viability was measured for IC50 studies using the CellTiter-Glo Luminescent Cell Viability Assay kit (Promega Inc., Madison, WI, USA) per manufacturer's instructions. (**B**) Normal human bone marrow cells from healthy donors were cultured in methylcellulose and treated with concentrations of BI- D1870 ranging from 2.5–10 μM. After 14 days, colonies were scored based on morphology. Cells from normal bone marrow were resuspended at 1×10^5 cells/mL in human StemMACS HSC-CFU media complete containing Epo (Miltenyi Biotec, Bergisch Gladbach, Germany) with concentrations of BI-D1870 ranging from 2.5–10 μM. After 14 days, colonies were counted as burst forming unit-erythroid (BFU-E), colony forming unit-granulocyte, monocyte (CFU-GM), colony forming unit-erythroid (CFU-E), or colony forming unit-granulocyte, erythrocyte, monocyte, macrophage (CFU-GEMM) based on morphology. Data are graphed as mean ± SEM (n = 3).

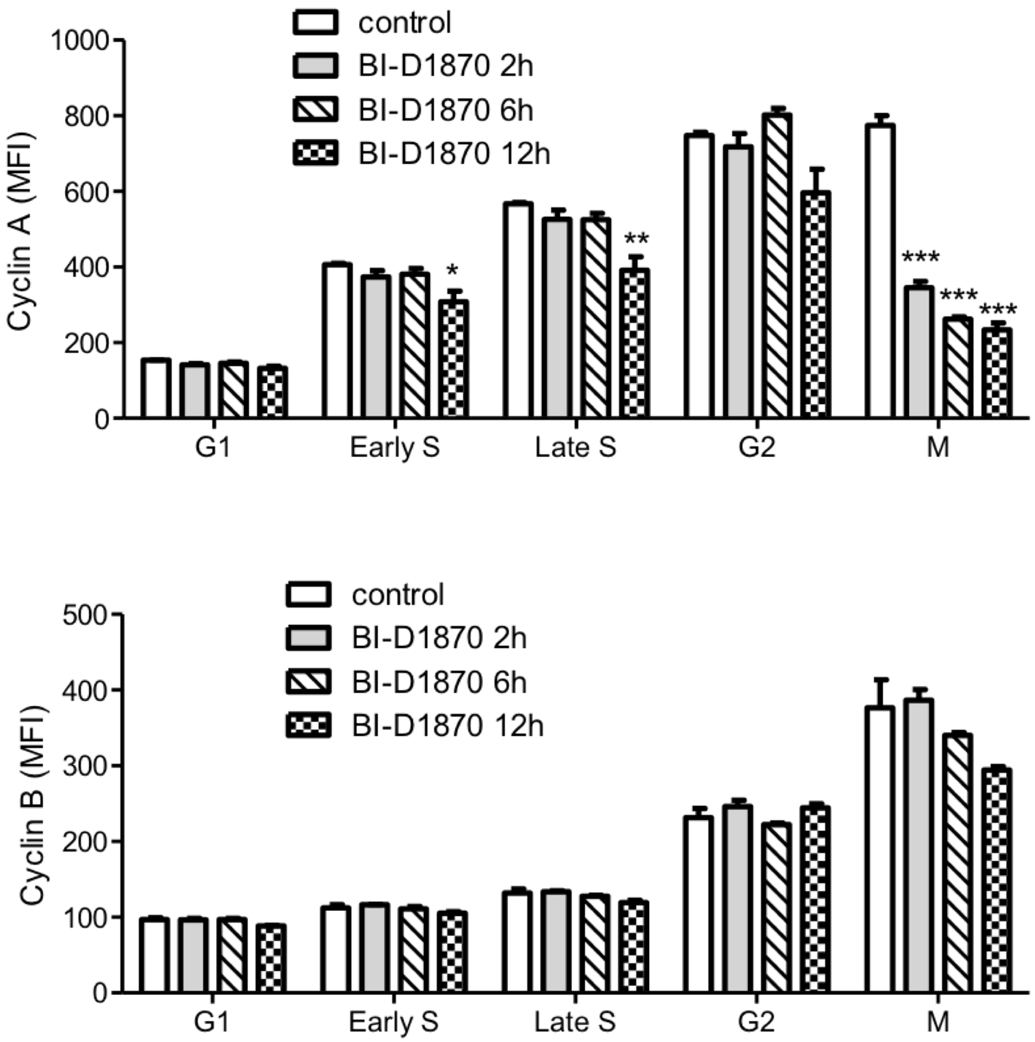

Supplementary Figure 3: Cellular levels of Cyclin A and Cyclin B in cell cycle phases. HL60 cells were treated with BI-D1870 (5 μM) and stained with DAPI and antibodies against p-H3, Cyclin A, and Cyclin B. Cellular levels of the Cyclin A (top) and Cyclin B (bottom) in each cell cycle phase were assessed by flow cytometry analysis. The graphs show median fluorescence intensities (MFI) of Cyclin A and Cyclin B in each stage. Values represent mean ± SEM ($n = 3$). $^{*}p < 0.05$; $^{**}p < 0.01$; $^{***}p < 0.001$.

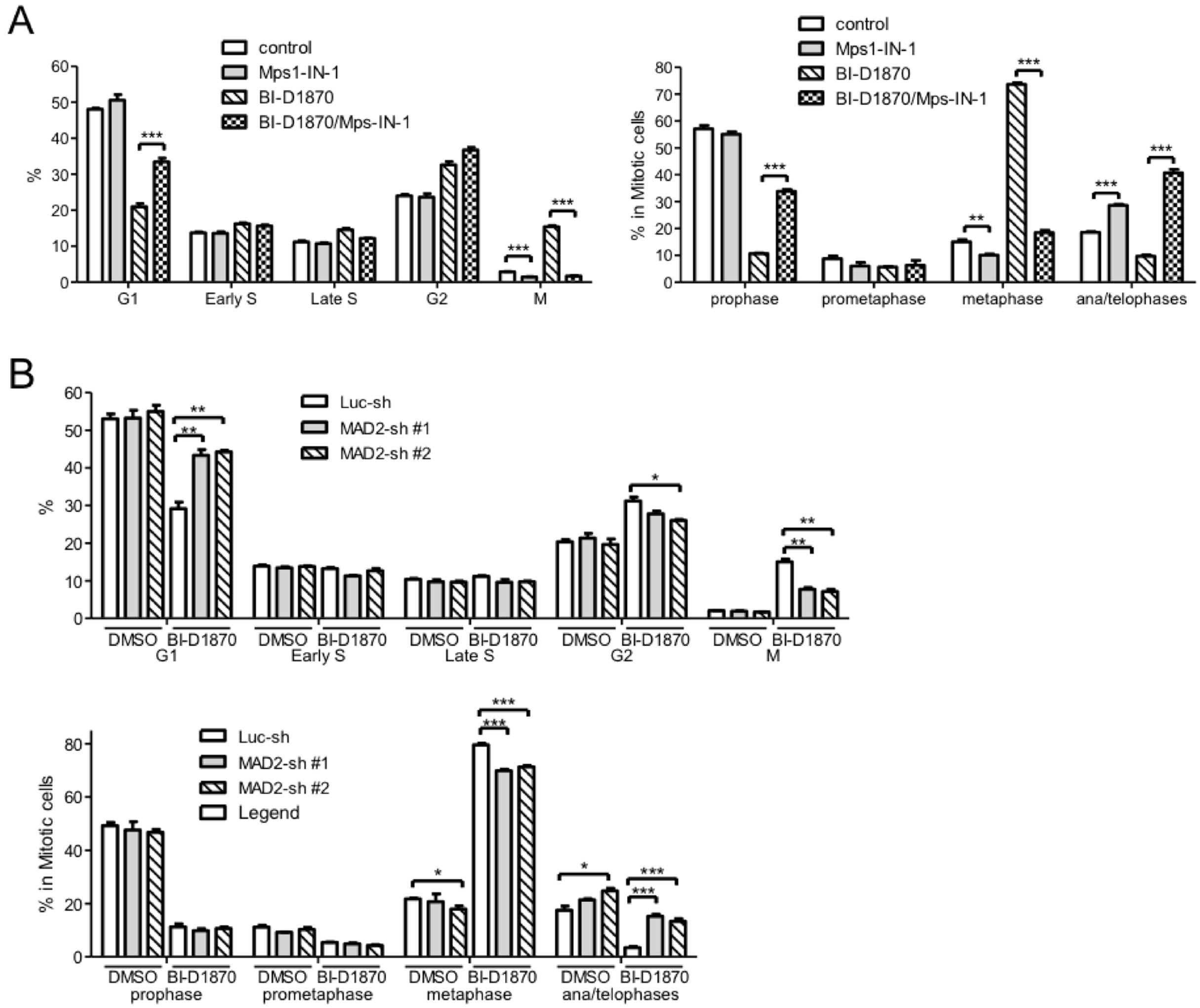

Supplementary Figure 4: Inactivation of SAC or MAD2 knockdown releases cells from BI-D1870-induced metaphase arrest. (**A**) HL60 cells were cultured with BI-D1870 (5 μM) or DMSO vehicle control for 12 h. Mps1 kinase inhibitor Mps-IN-1(10 μM) was added 90 min before collecting cells to assess the effect of SAC inhibition on BI-D1870-induced metaphase arrest. Cell populations on different cell cycle stages were plotted. (**B**) MAD2 knockdown alleviates the RSK inhibitor BI-D1870-induced metaphase arrest. MAD2 knockdown cells were treated with BI-D1870 (5 μM) for 12 hours, and then cells were fixed and analyzed for cell cycle distribution. Percentages of the cell population at each cell cycle stage were shown in graphs. Data are graphed as mean ± SEM (n = 3). $^{*}p < 0.05$; $^{**}p < 0.01$; $^{***}p < 0.001$.

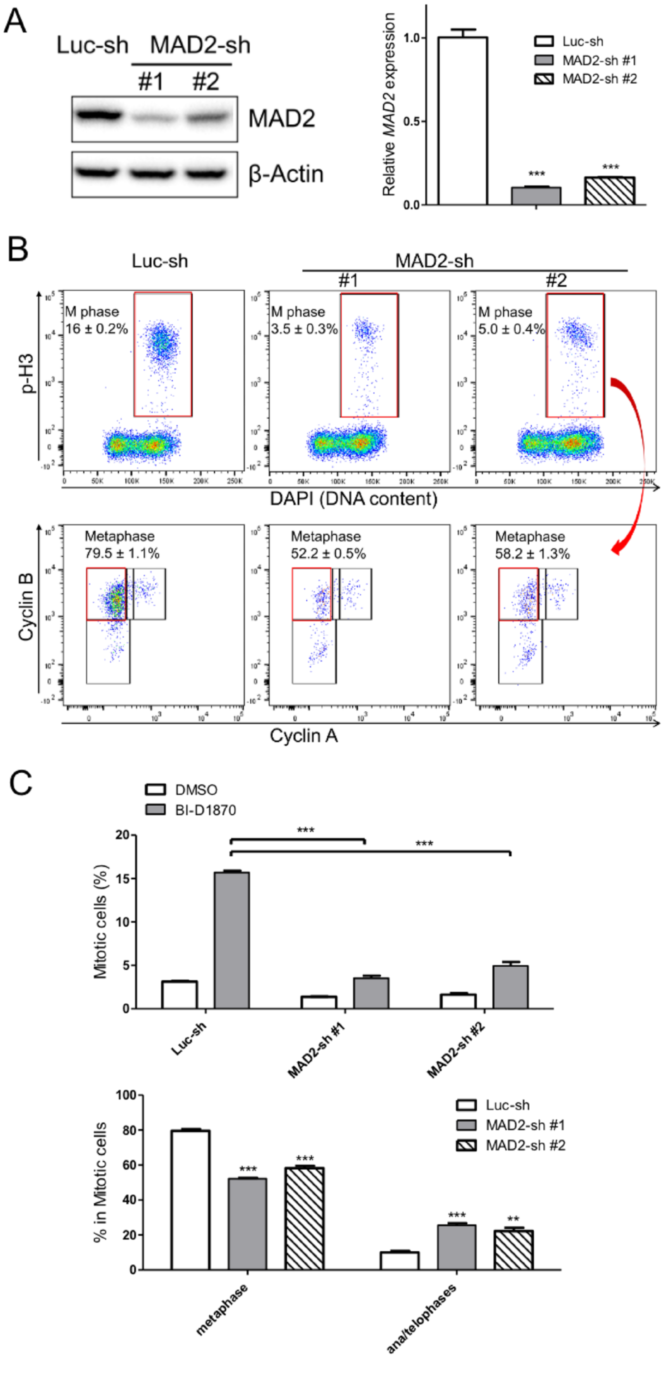

Supplementary Figure 5: RSK inhibitor BI-D1870-induced metaphase arrest is relieved in MAD2 knockdown KG1 cells. KG1 cells were infected with lentiviral vectors expressing MAD2 shRNA or luciferase shRNA. (**A**) Suppressed expression of MAD2 was confirmed at the protein level by immunoblotting and in mRNA levels by qRT-PCR. (**B**) KG1 cells expressing MAD2 or luciferase shRNA were treated with BI-D1870 (5 μM) for 16 hours. Levels of Cyclin A, Cyclin B, p-H3 and DNA contents were analyzed with flow cytometry. The mitotic cell population was determined by DNA content and protein levels of p-H3, Cyclin A, and Cyclin B (top). (**C**) Percentages of cell populations residing at M phase (top), or metaphase and ana/telophases phase (bottom) were calculated using FlowJo software and are expressed as mean ± SEM ($n = 3$). $^{**}p < 0.01$; $^{***}p < 0.001$.

Supplementary Table 1: Cytogenetics of AML patient samples

Total (n = 410)		
**Cytogenetics**	t(8;21)	15.9%
	inv(16)	13.9%
	Normal karyotype	27.8%
	t(9;11)(p22;q23)/11q23	17.6%
	–5, –7, or +8	7.8%
	Other	17.1%

Supplementary Table 2: Additive combination effect of BI-D1870 and chemotherapeutic drugs in HL60 cells

Daunorubicin			Cytarabine		
**CI Values at ED50**	**ED75**	**ED90**	**ED50**	**ED75**	**ED90**
0.951 ± 0.228	1.017 ± 0.091	1.092 ± 0.066	1.110 ± 0.057	1.149 ± 0.043	1.270 ± 0.069

Cells were treated with various concentrations of BI-D1870 and chemotherapeutic drugs for 3 days. Viability was assessed using the CellTiter glo assay kit. The combination index (CI) values were calculated by the Chou-Talalay method using CalcuSyn software. CI >1: antagonism, CI = 1: additive Effect, CI < 1: synergism. Data are the mean ± SEM (n = 4). Combinations of compounds, regulating the same target or same pathway, are reported to show additive combinatory effect.
